# Study of the Effectiveness of Alumina and HDTMA/Alumina Composite in the Removal of Para-Nitrophenol and the Deactivation of Bacterial Effect of *Listeria monocytogenes* and *Salmonella* spp.

**DOI:** 10.3390/life12111700

**Published:** 2022-10-26

**Authors:** Mustapha Aazza, Chadia Mounir, Hammou Ahlafi, Hamou Moussout, Aziz Bouymajane, Mounia Chroho, Filippo Giarratana, Luca Nalbone, Francesco Cacciola

**Affiliations:** 1 Laboratory of Chemistry-Biology Applied to the Environment, Faculty of Sciences, Moulay Ismail University, B.P 11201, Meknes 50070, Morocco; 2 Department of Veterinary Science, University of Messina, 98168 Messina, Italy; 3 Laboratory of Advanced Materials and Process Engineering, Faculty of Sciences, University IbnTofail, B.P 133, Kenitra 14000, Morocco; 4 Team of Microbiology and Health, Laboratory of Chemistry-Biology Applied to the Environment, Faculty of Sciences, Moulay Ismail University, B.P 11201, Meknes 50070, Morocco; 5 Molecular Chemistry, Materials and Catalysis Laboratory, Faculty of Sciences and Technologies, Sultan Moulay Slimane University, B.P 523, Beni-Mellal 23000, Morocco; 6 Department of Biomedical, Dental, Morphological and Functional Imaging Sciences, University of Messina, 98125 Messina, Italy

**Keywords:** adsorption, Al_2_O_3_, HDTMA^+^/Al_2_O_3_, para-nitrophenol, antibacterial activity

## Abstract

Removal of para-nitrophenol (p-NP) from an aqueous solution was studied under various batch adsorption experiments, using alumina (Al_2_O_3_) and its composite hexadecyltrimethylammonium bromide (HDTMA^+^-Br^−^) as adsorbents. These were later characterized, before and after adsorption of p-NP, by thermal analysis (DSC-TG), X-ray diffraction (XRD), Fourier transform infrared (FTIR), and UV/Visible spectroscopies. The results show that HDTMA^+^/Al_2_O_3_ adsorbents have a greater affinity toward p-NP than Al_2_O_3_ alone. Linear and non-linear forms of kinetics and isotherms were used to analyze the experimental data obtained at different concentrations and temperatures. The results indicate that the pseudo-second order kinetic model provided the best fit to the experimental data for the adsorption of p-NP on both adsorbents, and that the intra-particle diffusion was not only the rate controlling step. Both the Langmuir and Redlich-Peterson (R-P) models were found to fit the sorption isotherm data well, but the Langmuir model was better. Physical adsorption of p-NP onto the adsorbents proved to be an endothermic and spontaneous process at high temperatures, which mainly involves a hydrogen bonding mechanism of interactions between p-NP and functional groups of adsorbents. The antibacterial activity of Al_2_O_3,_ HDTMA^+^-Br^−^ and HDTMA^+^/Al_2_O_3_ were evaluated against *Listeria monocytogenes* and *Salmonella* spp. strains using both disc diffusion and broth microdilution methods. The HDTMA^+^-Br^−^ and HDTMA^+^/Al_2_O_3_ displayed a bacteriostatic effect against all tested strains of *Listeria monocytogenes* and *Salmonella* spp., while Al_2_O_3_ exhibited no bacterial effect against all bacterial strains tested.

## 1. Introduction

Due to the environmental risks resulting from the untreated industrial releases of various pollutants in wastewater, researchers are concerned about their elimination by suitable treatment methods to bring their concentrations to tolerated values [1,2]. Among these toxic pollutants, phenolic compounds, including nitrophenols, are a common pollutant found in wastewaters in large quantities, due to their use in several chemical and pharmaceutical manufacturing processes, such as herbicides, fungicides, insecticides, dyes, explosives, paints, and as solvents and precursors [3,4,5]. Given the toxicity of nitrophenolic compounds (ecotoxicity, mutagenicity, immunotoxicity, reproductive toxicity, and carcinogenic effects on the urinary system) [6,7], para-nitrophenol (p-NP) can cause, even at low concentrations, liver damage, anemia, tissue erosion, paralysis of the central nervous system, headaches and dizziness, and other related symptoms [8,9]. For these reasons, the effective removal of p-nitrophenol from liquid effluents continues to be the subject of much research, the objective of which is to meet the accepted environmental standards.

To achieve this goals, different treatment methods, such as coagulation/flocculation, membrane filtration [10], biological treatments [11], catalytic reduction [12,13], adsorption [14,15], advanced oxidation processes, etc., have been used; however, adsorption remains the most effective technic that can provide promising results for eliminating this type of pollutant, and has great benefits when using a cheaper adsorbent [16,17,18,19,20,21,22] than activated carbon, which is expensive and difficult to regenerate. In this sense, many cheaper alternative adsorbents have been employed to remove by adsorption the p-NP pollutant from liquid effluents [8,23,24,25]. As an example, natural clay and its various modified forms have been used successfully towards a given pollutant due to its low cost and high removal efficiency [8,25,26,27]. Moreover, in our previous works [14,15], the use of Al_2_O_3_ and its composite HDTMA^+^/Al_2_O_3_ as adsorbents in the adsorption of meta-nitrophenol (m-NP) and ortho-nitrophenol (o-NP) have shown promising results. Indeed, these adsorbents have many practical advantages, including abundant availability, low-cost, highest affinity toward nitrophenlic compounds and, in addition, the composite HDTMA^+^/Al_2_O_3_ is easy to obtain by a simple reaction between HDTMA^+^ polymer and Al_2_O_3_.

In the present work we report the use of these adsorbents to remove the para-nitrophenol (p-NP) from aqueous solutions and to evaluate their antibacterial properties through growth inhibition towards bacteria strains, such as *Listeria monocytogenes* and *Salmonella* spp. using disk diffusion and micro-dilution assays. These bacteria are known to cause food contamination [28]; therefore, preventing their growth by using these non-toxic materials will be beneficial to human health. To our knowledge. This study has not been reported in the literature yet.

Kinetics and isotherms experiments have been performed at different adsorption temperatures and explored to determine the thermodynamics parameters. The experimental data were modeled using various isotherm models (Langmuir, Freundlich, and Redlich-Peterson) to better understand the adsorption mechanism of p-NP adsorption. Before their use, the adsorbents were characterized by pH of the point of zero charge (pH_pzc_), XRD, FTIR, UV/Visible diffuse reflectance (DR) spectroscopy, and thermal (TGA/DSC) analysis.

## 2. Materials and Methods

### 2.1. Material

The para-nitrophenol (p-NP: HOC_6_H_4_NO_2_) used was reagent grade provided by Aldrich Chemicals, while (γ-Al_2_O_3_, LR) was from Lobachemie (Inde). Hexadecyltrimethyl ammonium bromide (CH_2_)_15_(CH_3_)_3_N^+^Br^−^ (HDTMA^+^-Br^−^) was obtained from Fluka. All of these chemicals were analytical grade (99% purity), and were used as received without further purification. The test solutions were prepared by diluting the stock solution in bi-distilled water to obtain the desired concentrations of p-NP in the range of 0.02 to 0.2 mM. Other required reagents, such as HCl and NaOH, were used to adjust the pH of the solutions; these were analytical grade and purchased from Aldrich Chemicals.

### 2.2. Preparation of HDTMA^+^(xM)/Al_2_O_3_ Composite

Here, a HDTMA^+^(0.1 M/Al_2_O_3_) composite was synthesized according to the reported procedure [14]. Briefly, a mass m(g) of Al_2_O_3_ was dispersed in a volume V(mL) of HDTMA^+^-Br^−^ solution (0.1 M) and stirred for 24 h at room temperature. The composite was then separated from the liquid phase by centrifugation at 4500 rpm and washed thoroughly several times with bi-distilled water until complete removal of Br^−^ ions from the solid was obtained (test with silver nitrate (Ag(NO_3_)). Finally, the composite was dried for 6 h at T = 60 °C and then ground in an agate mortar, before the powder was stored for further use.

### 2.3. Characterization

To determine the changes in the sample (Al_2_O_3_) and the HDTMA^+^(0.1 M)/Al_2_O_3_) composite, before and after their contact with p-NP, different spectroscopic techniques were used, as follows:

- Fourier transform infrared spectra (FTIR) were collected from 400 to 4000 cm^−1^ at a resolution of 4 cm^−1^ on an FTIR spectrometer (Shimadzu, JASCO 4100) using a KBr pellet technique. The samples were first ground and then mixed with KBr at a w/w: 4/96 ratio of (sample/KBr).
- UV/Visible spectra of fine powder samples of Al_2_O_3_ and HDTMA^+^/Al_2_O_3_ composite and p-NP solutions were recorded using a JASCO V-750 spectrophotometer equipped with an integrating sphere using quartz window, whereas, p-NP in the solutions, which reach a maximum absorption band at λ = 396 nm, was quantified during the adsorption experiments using a standard quartz cuvette.
- Differential scanning calorimetry measurements (DSC) of samples were performed using a DSC 131 Evo instrument and thermogravimetric analysis (TGA) was carried out with Shimadzu TA 60 instrument in the same experimental conditions. Samples were placed in alumina cup and then heated at 20 °C/min from room temperature to 600 °C under air atmosphere. The corresponding empty cup of alumina was used as reference. Heat flux and mass loss are recorded as a function of degradation temperature.

### 2.4. Method

#### 2.4.1. Kinetics and Isotherms

The kinetics and isotherms adsorption experiments of p-NP onto alumina and HDTMA^+^/Al_2_O_3_ composite were performed using the batch contact method. Kinetic experiments were conducted with a mass m = 0.1 g of the adsorbent contacted with V = 20 mL of the p-NP solution of concentration C_0_ = 0.4 mM in sealed flask and agitated at 600 rpm during a predetermined time (t) of adsorption at a constant temperature (T = 25, 35 and 45 °C) maintained with a thermostatic bath. In all the experiments, the pH value was adjusted at pH = 6 using an appropriate concentration of HCl or NaOH solutions. The choice of working pH = 6 is based on our previous study of the adsorption of nitrophenlic compounds [14,15]. After each contact time t, the mixture was filtered through a membrane filter (0.45 µm pore size) and the residual concentration C_e_ of p-NP left in the solution was determined by a UV/Visible spectrophotometer at λ = 396 nm, which corresponds to the maximum absorption wavelength of p-NP. The adsorption capacity of the adsorbent was determined by material balance of the initial and equilibrium concentrations of p-NP solution, using the following equation:

$$q_{t}=\frac{C_{0}-C_{res}}{m_{ads}}V_{\mathrm{sol}}$$

where q_t_ is the adsorption capacity of adsorbents (mg/g), V(L) is the volume of solution, and C_0_ is the initial concentration of p-NP.

The adsorption equilibrium isotherms of p-NP onto the adsorbents were performed in the same experimental conditions as those of kinetic experiments by varying the initial concentration of p-NP in the range of 0.04 to 0.4 mg/L.

Experimental kinetic and isotherm data obtained at different temperatures were subjected to different linear and nonlinear models given, and the validities of each of them were confirmed by the error analysis calculations (Table 1). Then, the corresponding parameters were determined.

#### 2.4.2. Antibacterial Activity

The bacterial strains of *Listeria monocytogenes* and *Salmonella* spp. were obtained from the Laboratory of Microbiology and Health at our university. Bacterial strains were cultured at 37 °C for 24 h on tryptone soya yeast extract agar (TSYEA) (Biolife, Milan, Italy). Then, bacterial suspensions were prepared in a sterile physiological solution of NaCl (0.9%), and then adjusted to the equivalent of 0.5 McFarland standards (10^8^ cfu/mL).

##### Disc Diffusion Method

The disc diffusion method was used to preliminarily test the antibacterial activity of the samples (HDTMA^+^-Br^−^, Al_2_O_3_, and the HDTMA^+^/Al_2_O_3_ composite) against different strains of *Listeria monocytogenes* and *Salmonella* spp. Briefly, 5 µL of different concentrations of HDTMA^+^-Br^−^, Al_2_O_3_, and HDTMA^+^/Al_2_O_3_ composite prepared in TSYEA (0.05, 0.1, 0.5, 1 and 5 mg/mL) was dropped on 6 mm diameter sterile paper discs (Biolife, Milan, Italy).

##### Microdilution Method

The minimum inhibitory concentration (MIC) and minimum bactericidal concentration (MBC) of the samples (HDTMA^+^-Br^−^; Al_2_O_3_ and HDTMA^+^/Al_2_O_3_ composite) against *Listeria monocytogenes* and *Salmonella* spp. strains were evaluated by the microdilution method, as described by Bouymajane et al. [29] with some modifications. In flat-bottom 96-well microplates, 80 µL of different concentrations of the materials prepared in TSYEB (0.05, 0.1, 0.5, 1, and 5 mg/mL) were first added to 10 wells. Afterward, 20 µL of bacterial suspension was added to each well. The well containing bacterial suspension with TSYEB and the well containing the materials prepared in TSYEB were considered as a positive and negative controls, respectively. After microplate incubation at 37 °C for 24 h, 10 µL of TTC (2,3,5,-triphenyl tetrazolium chloride) was added to each well and re-incubated at 37 °C for 30 min. The MIC was determined at the lowest concentration of materials at which the bacterial growth was not observed. On the other hand, MBC was determined at the lowest concentration of materials that did not produce any bacterial colony, by plating 10 µL of samples from the wells in which no bacterial growth was observed on the TSYEA. The r = MBC/MIC ratio is used to determine the bacteriostatic and bactericidal effects of the materials; if the r ratio is below than 4, the effect is bactericidal, if the r ratio is greater than 4, the effect is bacteriostatic.

The antibacterial activity was carried out at the Microbiology Laboratory of the University of Messina in Italy, as part of Erasmus^+^ Mobility.

**Table 1:** Kinetics, isotherms, and thermodynamics equations for the adsorption of p-NP on the adsorbents.

Kinetic Models		Ref
Pseudo-first order	$\ln(q_{e}-q_{t})=\ln(q_{e})-k_{1}t$	[30]
Pseudo-second order	$\frac{t}{q_{t}}=\frac{1}{k_{2}q_{e}^{2}}+\frac{1}{q_{e}}t$	[31]
Weber and Morris	$q_{t}=k_{\mathrm{int}}\sqrt{t}+C_{i}$	[32]
**Isotherms models**		
Langmuir	$q_{e}=\frac{q_{m}K_{L}C_{e}}{1+K_{L}C_{e}}$	[33]
Freundlich	$q_{e}=K_{F}C_{e}^{1/n}$	[34]
Redlich-Peterson	$q_{e}=\frac{AC_{e}}{1+K_{R}C_{e}^{\alpha }}$	[35]
**Thermodynamic equations**		
Activation energy	$Ln(k_{2})=Ln(A)-\frac{E_{a}}{RT}$	
Standard thermodynamic parameters	$\ln(K_{c})=-\frac{\Delta H^{\circ }}{RT}+\frac{\Delta S^{\circ }}{R}$	[36]
	$\Delta G^{\circ }=-RT\ln(K_{C})$	
**Error functions**		
SD: Normalized standard deviation	Δq(%)=100×{∑i=1n[(qexp−qcal)qexp]2n−1}1/2	[37,38]
χ^2^: Chi-square analysis	$\chi^{2}=\sum_{i=1}^{n}\frac{{\left(q_{exp}-q_{cal}\right)}^{2}}{q_{cal}}$	
R^2^: Coefficient of determination	$R^{2}=\frac{\sum_{i=1}^{n}{\left(q_{cal}-{\bar{q}}_{exp}\right)}^{2}}{\sum_{i=1}^{n}{\left(q_{cal}-{\bar{q}}_{exp}\right)}^{2}+\sum_{i=1}^{n}{\left(q_{cal}-q_{\exp}\right)}^{2}}$	

Where q_m_ is the adsorbed amount at equilibrium (mg/g), C_e_ (mg/L) is the residual concentration at equilibrium, and K_L_ (L/mg) represents the ratio of the rate constant of adsorption and desorption. Here, k_d_ and C are the intra-particle diffusion rate constant and thickness of the boundary layer, respectively, while K_F_ and n are Freundlich constants that express the capacity and the adsorption intensity, respectively. Furthermore, A_RP_ and K_RP_ are the Redlich-Peterson isotherm constants and α (0 < α < 1) is the exponent reflecting the heterogeneity of the adsorbent, while A is the preexponential factor (min^−1^); K_c_ is the equilibrium constant, while C_ads_ is the amount of p-NP adsorbed on the adsorbent of the solution at equilibrium (mg/L), and T is the absolute temperature (K).

### 2.5. Determination of the Point of Zero Charge: Pzc

The point of zero charge corresponds to the pH(pzc) where the net number of positive and negative charges are equal and, thus, the total surface charge becomes zero. The pH_pzc_ of Al_2_O_3_ and HDTMA^+^/Al_2_O_3_ were determined using the volumetric potentiometric titration method developed recently in our laboratory and detailed in Reference [39]. The potentiometric titrations are carried out at 298 K in a special thermostated cell in the presence of the solids in the solution and in their absence (blanks). Briefly, the procedure is as follows: a mixture of V = 100 mL of NaCl electrolyte (C = 10^−3^ mol/L) and V = 1 mL of HCl (C = 0.50 mol/L) is titrated gradually by adding a volume of 50 ± 0.005 µL of NaOH solution (C = 0.2 M) to the mixture. The assay is stopped after each addition of NaOH when there is no variation in the pH final (pH_f_) of the solution. Then, the density of surface charge (σ_0_) is calculated as a function of pH_f_. The titrations of solids are carried out in the same way as in their absence.

## 3. Results and Discussion

### 3.1. Characterization of the Adsorbents

#### 3.1.1. UV/Visible

**Figure 1:**
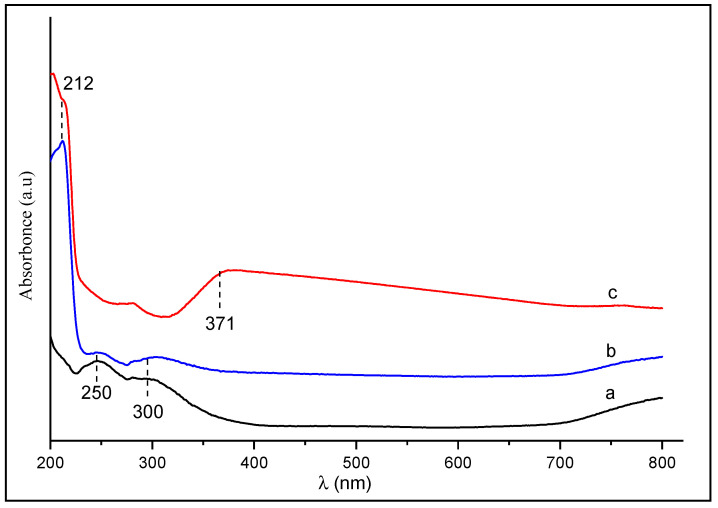
UV/Vis spectra of (**a**) Al_2_O_3_, (**b**) HDTMA^+^/Al_2_O_3_, and (**c**) HDTMA^+^-Br^−^.

The UV/Vis spectra of Al_2_O_3_, HDTMA^+^ and HDTMA^+^/Al_2_O_3_ composite are shown in Figure 1. In the absence of the polymer, two characteristic bands of γ-Al_2_O_3_ are observed at λ = 250 nm and λ = 300 nm (spectrum a). These bands keep their initial positions and decrease in intensities in the spectra (b) of HDTMA^+^/Al_2_O_3_, due to the adsorption of HDTMA^+^ on the alumina surface. The appearance of a new band at λ = 212 nm can be attributed to electronic transitions of σ → σ*, characteristic of saturated hydrocarbons. However, the wide band observed at λ = 371 nm in the spectra (b) of HDTMA^+^ corresponds to n → σ* transitions. This band does not appear in the spectrum (c) of the composite, probably because the original electrons are engaged in the interactions between the polymer and alumina.

#### 3.1.2. Thermogravimetric Analysis: TGA/DrTGA

**Figure 2:**
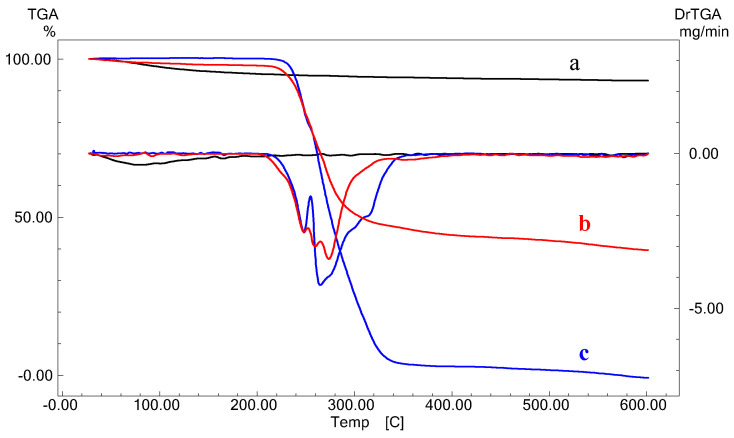
TGA/DrTGA thermograms of (**a**) Al_2_O_3_, (**b**) HDTMA^+^/Al_2_O_3_, and (**c**) HDTMA^+^-Br^−^.

The TGA/DrTGA thermograms of Al_2_O_3_ and the HDTMA^+^/Al_2_O_3_ composite recorded under an air atmosphere are given in Figure 2. It can be seen that the degradation of HDTMA^+^/Al_2_O_3_ is slower than that of the initial HDTMA^+^ polymer. In the temperature range studied, the total mass loss of the composite is (−59.54%), lower than that of HDTMA^+^ (−100%), which indicates that HDTMA^+^ molecules are strongly bounded to alumina, thus, forming the corresponding composite. The three peaks observed in the DrTGA curves of the two solids, ranging from T = 240 °C to T = 280 °C, are attributed to the different stages of decomposition and oxidation of the polymer HDTMA^+^ [40]. The endothermic peak observed at T = 77 °C in all thermograms is due to the physisorbed water.

#### 3.1.3. DSC

**Figure 3:**
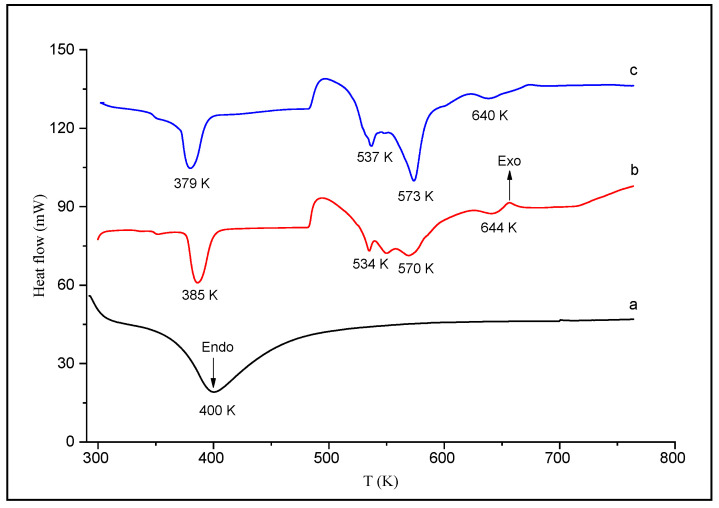
DSC curves of (**a**) Al_2_O_3_, (**b**) HDTMA^+^-Br^−^, and (**c**) HDTMA^+^/Al_2_O_3_.

The samples were also studied using DSC analysis in the same conditions as the TGA experiments. Figure 3 shows that the first endothermic peak is attributed to the loss of surface water, and the subsequent peaks are due successively to the different stages of decomposition and oxidation of the polymer. It can be noticed that for T > 500 K, the peaks observed for HDTMA^+^/Al_2_O_3_ are not similar to those obtained for HDTMA^+^ alone, which confirms the interaction of HDTMA^+^ with Al_2_O_3_ in accordance with previous TGA curves.

#### 3.1.4. Point of Zero Charge

**Figure 4:**
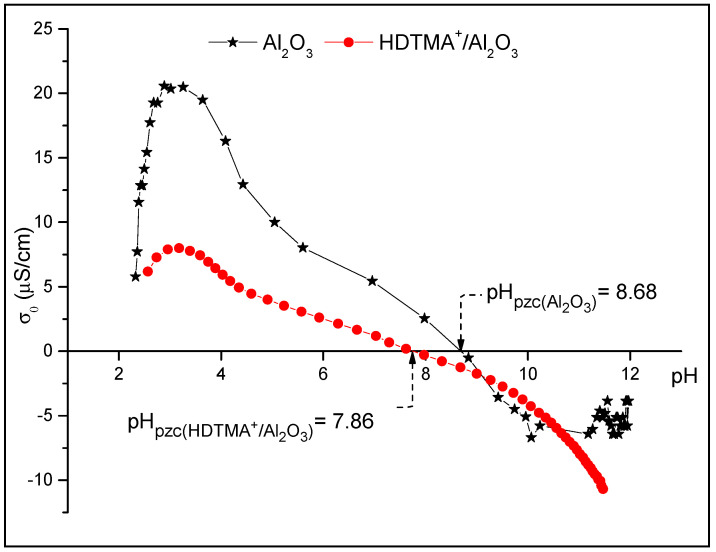
Potentiometric titration of Al_2_O_3_ and HDTMA^+^/Al_2_O_3_ in NaCl at T = 25 °C.

Figure 4 shows the curves of σ_0_ as a function of pH_f_, obtained using the potentiometric titration method. The pH_pzc_ of the alumina and the composite are at pH = 8.3 and 7.7, respectively. These values are in good accordance with those found in the literature, based on mass [14,15] and zeta potential [41] measurements. Therefore, the surface charge of Al_2_O_3_ is positive at pH < 8.3 and that of HDTMA^+^/Al_2_O_3_ is positive at pH < 7.7. Above these pH values, the surface charges are respectively negative. Wisniewska et al. [42] found that the adsorption of the polymers on alumina leads to the reduction of zeta potential value in the solid particles, compared to alumina alone, due to more developed conformation of the adsorbed polymer chains on the solids, which increases the acidic groups in the composite.

### 3.2. Adsorption Kinetics

**Figure 5:**
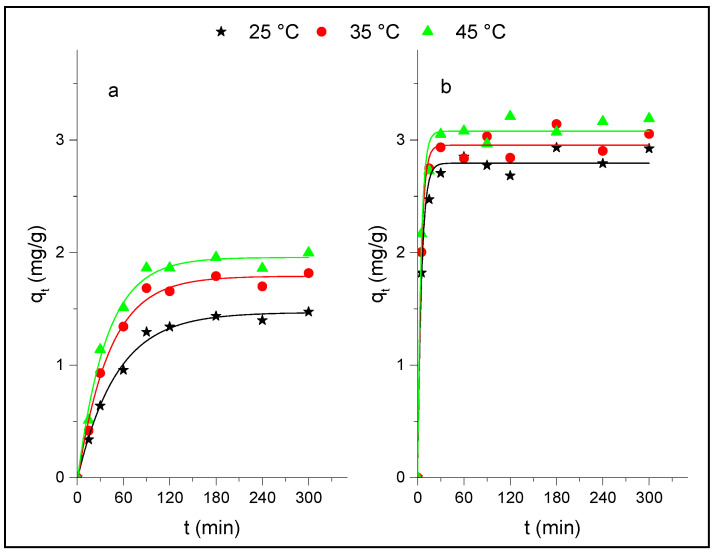
Experimental and modeled kinetic data of p-NP adsorption at different temperatures onto (**a**) Al_2_O_3_ and (**b**) HDTMA^+^/Al_2_O_3_ (m = 0.1 g, C_0_ = 0.4 mM, and pH = 6).

The study of adsorption as a function of time makes it possible to obtain information about the kinetic parameters involved in the process. Figure 5 shows the obtained kinetic curves for p-NP adsorption onto Al_2_O_3_ and HDTMA^+^/Al_2_O_3_ at T = 25, 35 and 45 °C, and at a constant pH = 6. These kinetic curves clearly showed that initial adsorption of p-NP on the composite occurred quickly during the first contact time (t < 30 min) at all temperatures studied, compared to its adsorption on alumina (t > 60 min). After these times, the removal of p-NP does not show a noticeable evolution, which indicates that the equilibrium times are reached. In addition, it is noted that the equilibrium uptake of p-NP onto the two adsorbents increases with the adsorption temperature and at the same temperature the adsorption capacity of the composite is greater than that obtained in the case of Al_2_O_3_ alone, due to the presence of HDTMA^+^ on the surface of Al_2_O_3_, which gives the composite a large number of sites available for the adsorption of p-NP. For example, for T = 45 °C, the adsorbed amounts of p-NP at equilibrium are q_e_ = 3 mg/g and 2 mg/g for the composite and alumina, respectively. This behavior can be related to the nature of the interaction involved in the adsorption process. Indeed, at pH = 6, p-NP exists in the solution in its neutral form and is less ionized to phenolate, given its pK_a_ value (pK_a_ = 7.15), and the adsorbent surfaces are always positively charged (pH_pzc_ > 6). Moreover, it has been observed that p-NP does not adsorb at pH > 7 [43,44,45,46], which suggests that different forces other than the electrostatic forces can be involved in the adsorption of p-NP. It was indicated that the π-π stacking interaction between benzene rings of nitrophenol and the surface of the adsorbents might be the plausible route for the adsorption of p-NP. Furthermore, a comparison of the adsorbed amounts of p-NP at equilibrium with those obtained in our previous study for the adsorption of ortho-NP and meta-NP isomers on these solids, under the same experimental conditions [14,15], shows that p-NP adsorbs less (o-NP > m-NP > p-NP), indicating that the polarity of nitrophenlic compounds, which depends on the position of the nitro groups. This suggests that they also play an important role in their adsorption. Other authors [47,48], have found that molecular p-NP can be adsorbed onto graphene oxide at a lower pH by hydrophobic and π-π interactions with the aromatic moieties of the p-NP and the aromatic matrix of the graphene. This mechanism corresponds to the well-known donor−acceptor complexes. The increases in the adsorbed amount of p-NP onto HDTMA^+^/Al_2_O_3_ could also combine the nitrophenols through the hydrogen bonds originating from the amino groups [49]. However, the removal efficiency of p-nitrophenol increased as the temperature increased, indicating that the adsorption of p-NP on both solids is endothermic, which may be due to the changes in the pore structure of the adsorbent and/or the diffusive mass transfer.

**Figure 6:**
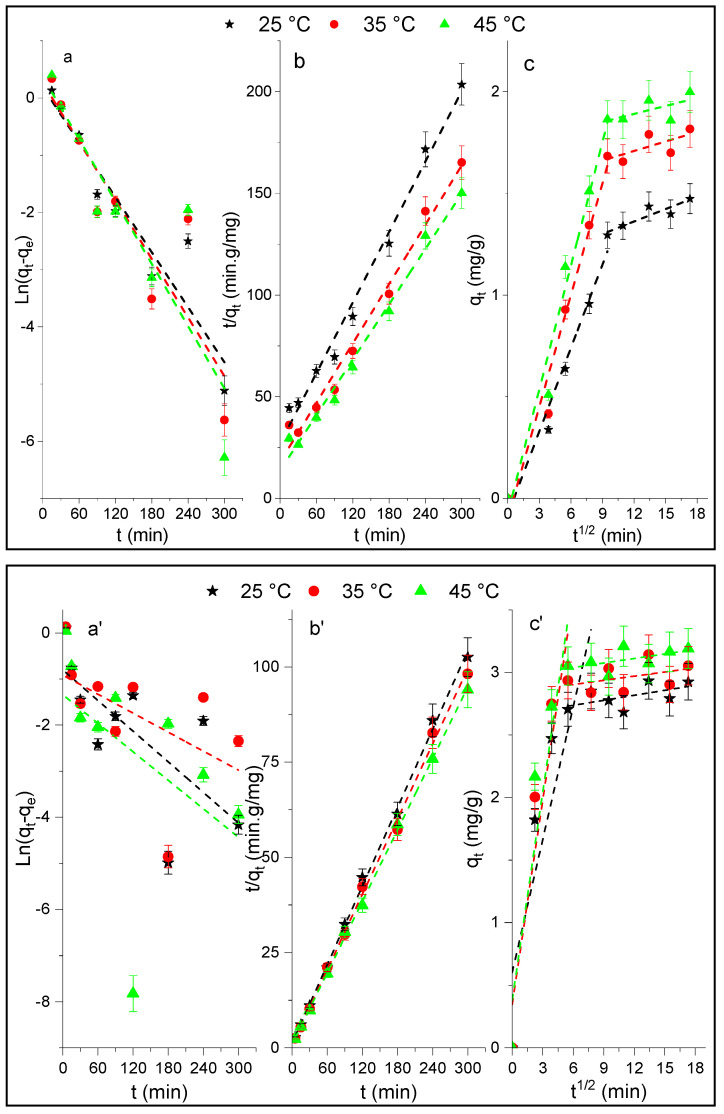
LPFO and LPSO intra-particle diffusion models for the adsorption kinetics of p-NP onto (**a**–**c**) Al_2_O_3_ and (**a’**–**c’**) HDTMA^+^/Al_2_O_3_ at different temperatures.

In order to obtain information regarding the kinetic parameters and mechanism of the p-NP adsorption onto Al_2_O_3_ and HDTMA^+^/Al_2_O_3_, experimental points given in Figure 5 were fitted using the linear kinetic models of pseudo-first order, pseudo-second order, and intra-particle diffusion. Figure 6 presents the corresponding plots, respectively, and the calculated kinetic parameters are given in Table 2. Figure 6(a,a’) showed that the linear pseudo-first order (LPFO) kinetics do not provide a straight line in the ranges of time and temperatures studied for both adsorbents, unlike the pseudo-second order (LPSO) model (Figure 6(b,b’)), which allows for a better fit of the experimental data. The validity of this model is confirmed by a good agreement obtained between the calculated and experimental values of q_e_, and the accuracies of different kinetic parameters (values of R^2^, Δq, and χ^2^ in Table 2). In addition, using the nonlinear equation (q_e_ vs. t) of this model (NLPSO) also leads to a good representation of the experimental data (curves in Figure 4). The values of rate constants k_2_ were found to increase as the adsorption temperature increases.

Moreover, the plotting of q_t_ vs. t^1/2^ leads to two linear parts with different slopes (Figure 6(c,c’)), which correspond to two adsorption mechanisms controlling the adsorption of p-NP onto Al_2_O_3_ and HDTMA^+^/Al_2_O_3_, and was not only controlled by the intra-particle diffusion. The results show that the diffusion rate constants (k_d1_) are greater than that of (k_d2_), indicating that in the first period, the molecules diffuse easily in the internal structure of the adsorbents, and then after the number of pores available for diffusion decreases. This reduces the free passage of molecules through the pores and some molecules may be blocked [50].

### 3.3. Adsorption Isotherm

**Figure 7:**
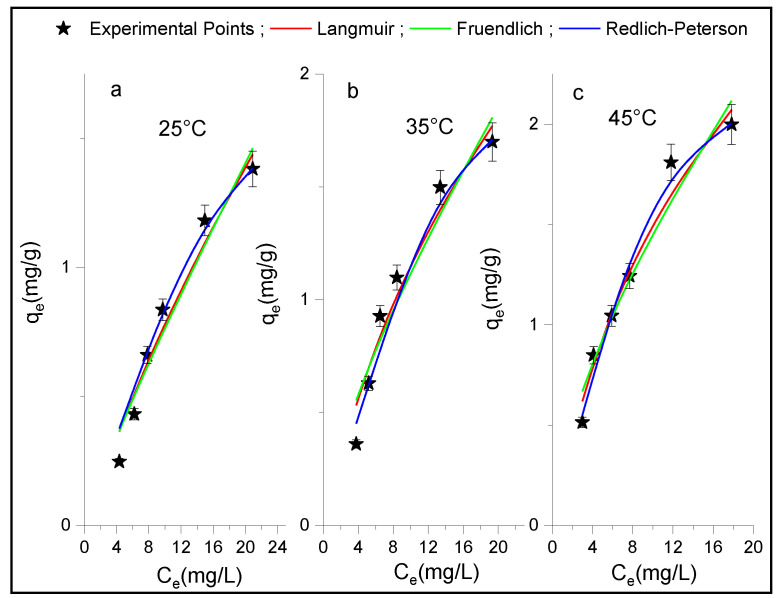
Adsorption isotherms of the p-NP on Al_2_O_3_ at (**a**) T = 25 °C, (**b**) T = 35 °C and (**c**) T = 45 °C plotted at each temperature with non-linear regression of the Langmuir, Freundlich, and Redlich-Peterson models.

**Figure 8:**
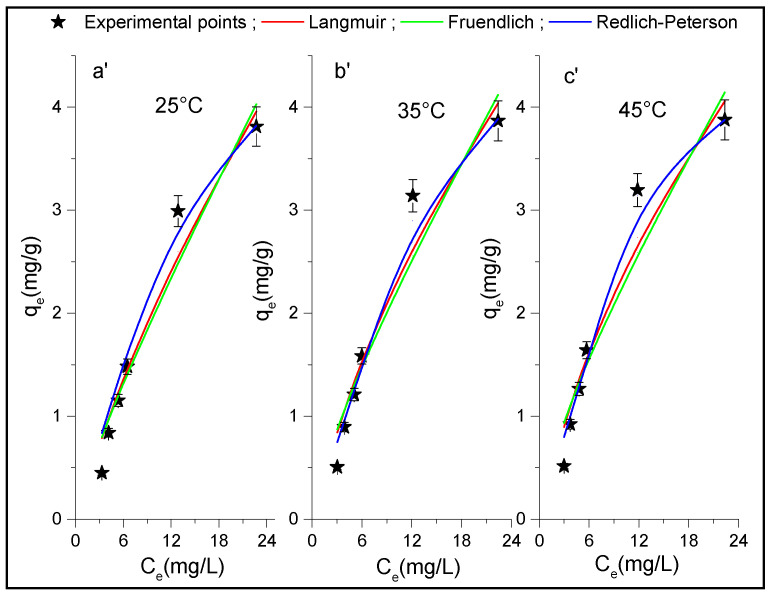
Adsorption isotherms of the p-NP on HDTMA^+^/Al_2_O_3_ at (**a’**) T = 25 °C, (**b’**) T = 35 °C and (**c’**) T = 45 °C plotted at each temperature with non-linear regression of the Langmuir, Freundlich, and Redlich-Peterson models.

Adsorption isotherms of p-NP onto Al_2_O_3_ and HDTMA^+^/Al_2_O_3_ were carried out at different temperatures and concentrations for an equilibrium adsorption time t = 3 h. The experimental isotherms data obtained and shown in Figure 7 and Figure 8 were fitted using nonlinear equations of the Langmuir, Freundlich, and R-P isotherms models. The calculated parameters from these plots along with the corresponding values of R^2^ and χ^2^ are summarized in Table 3. These can provide information about the monolayer and multilayer adsorption of adsorbate on surface sites of energetically homogeneous or heterogeneous adsorbents. A priori, it can be observed that the theoretical R-P curves seem to better follow the experimental isotherms data than those obtained with other models used (Figure 7 and Figure 8), because the R-P isotherm exhibited lower values of χ^2^ and higher values of R^2^ than the Freundlich and Langmuir models (Table 3) at any adsorption temperature for both adsorbents. However, based on the values of α, which must lie between 0 and 1, the R-P model only conforms to the results obtained in the case of the composite HDTMA^+^/Al_2_O_3_. It is known that the R-P model with three parameters can represent either the Langmuir or the Freundlich model, when α = 1 and 0, respectively. Thus, these limit values of α were not obtained in our case, and are much greater than 1 for alumina, suggesting that the R-P model is not suitable for describing the adsorption isotherms of p-NP on this adsorbent. On the other hand, taking into account the values of R^2^, the Langmuir isotherm can also be considered better for both adsorbents than the Freundlich model, which indicates that the adsorption of p-NP on these adsorbents take place at energetically homogeneous sites forming a molecular monolayer. In addition, it was reported that Freundlich isotherm is a special case of the R-P isotherm when constants A_R-P_ and K_R-P_ are much greater than unity [51]. Otherwise, the values of 1/n_F_ in the Freundlich isotherm are between 0 and 1 for all the temperatures studied, which indicates that the adsorption of p-NP was carried out easily.

### 3.4. Thermodynamic Study of p-NP Adsorption

**Figure 9:**
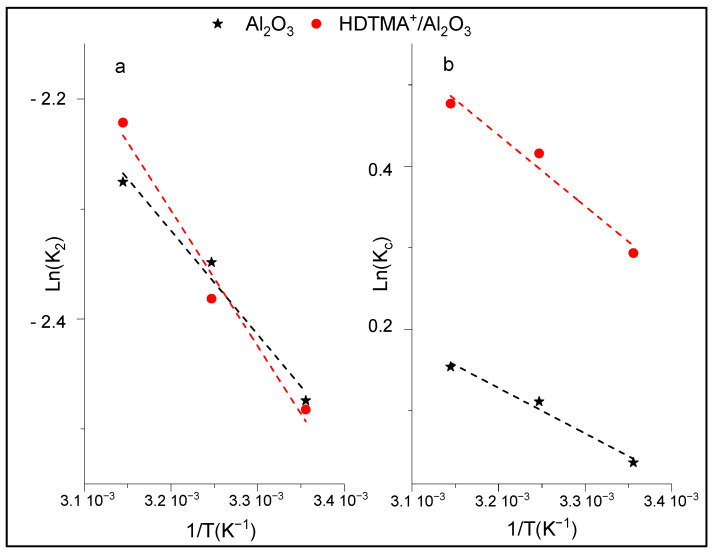
Arrhenius plots of p-NP adsorption on Al_2_O_3_ and HDTMA^+^/Al_2_O_3_.

The thermodynamic parameters, such as activation energy (E_a_), Gibbs free energy (ΔG°), enthalpy change (ΔH°), and entropy change (ΔS°), are useful to determine the spontaneity of p-NP adsorption onto Al_2_O_3_ and HDTMA^+^/Al_2_O_3_. The activation energy (E_a_) was evaluated from the slope of the linear form of the Arrhenius equation (Table 1), which expresses the pseudo-second order rate constant K_2_ as a function of adsorption temperature. According to Figure 9a, a linear plots of ln(K_2_) vs. (1/T) were obtained for both adsorbents in the temperature range studied (283–313 K). The activation energies of p-NP adsorption are E_a_ = 8 kJ/mol and E_a_ = 10 kJ/mol for Al_2_O_3_ and HDTMA^+^/Al_2_O_3_ (Table 4), respectively, indicating that the adsorption is an endothermic process, and likely dominated by physical adsorption (E_a_ < 40 kJ/Mol), involving weak attraction forces. Other considered thermodynamic parameters were also determined by plotting the linear form of the van’t Hoff equation (ln(K_c_) vs. (1/T) (Figure 9b), which led to the values of ∆H° and ∆S°, as listed in Table 4. Thus, the positive values of ∆H° (0 < ∆H° < 10 kJ/mol) and ∆S° show that the adsorption of p-NP on the adsorbents is endothermic and that the adsorbed species are disordered at the solid-liquid interface. The values of ∆G° were negatives, indicating that the adsorption of p-NP is spontaneous at all temperatures studied. This finding confirmed the previous results, mainly the increase in the p-NP adsorption capacities of adsorbents as the temperature increased, as well as the physical nature of the adsorption process. Additionally, the values of ∆H° indicated that p-nitrophenol has more energy when adsorbed onto HDTMA^+^/Al_2_O_3_ compared to Al_2_O_3_.

### 3.5. Adsorption Mechanism

**Figure 10:**
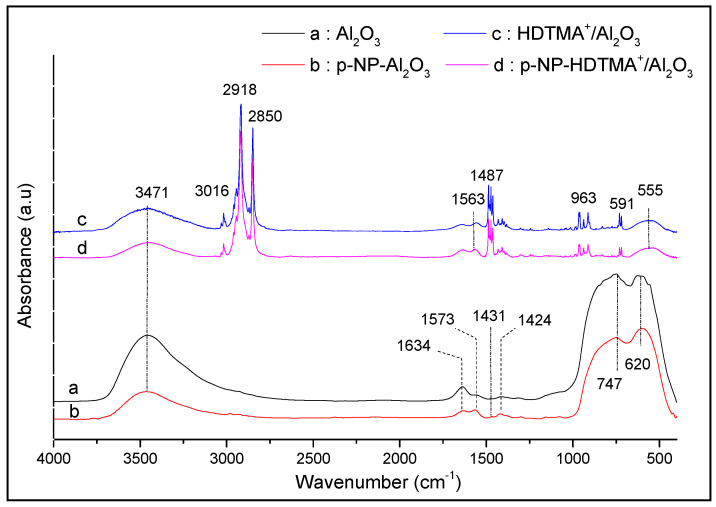
FTIR spectra of (**a,b**) Al_2_O_3_ and (**c,d**) HDTMA^+^/Al_2_O_3_ before and after adsorption of p-NP.

Based on the literature data, it appears that the adsorption of p-NP on different adsorbents is influenced by several factors. Among them is the nature of interactions of p-NP/Al_2_O_3_ and p-NP/HDTMA^+^/Al_2_O_3_, which can explain the differences obtained between their adsorption capacities. In this study, FTIR and XRD technics were used to characterize the changes in the functional groups of each adsorbent, after the adsorption of p-NP. Firstly, as can be seen in the FTIR spectrum of the composite HDTMA/Al_2_O_3_ (spectrum c, Figure 10), we observed the appearance of intense bands at 3016, 2918, and 2850 cm^−1^ (-CH_3_ et -CH_2_ vibrations), and at 1485 cm^−1^ (-N(CH_3_)_3_ vibration) due to the HDTMA^+^ polymer, while those of the initial bands of the alumina at 3471, 690, and 753 cm^−1^, attributed to the vibrations of Al-OH, Al-O, and O-Al-O, respectively, undergo a significant diminution. In addition, the positions of the last two bands shift towards low frequencies to give a single band at 555 cm^−1^. These different variations express a strong interactions between the alumina surface and the cationic HDTMA^+^ polymer, leading to the formation of the corresponding composite [14,15]. Furthermore, it is observed in the FTIR spectra (Figure 10) that after contact of p-NP with alumina, new bands appear, and the intensities of the bands related to O-H groups in the alumina decrease. This decrease can be related to the interactions of p-NP molecules in an acidic medium with the O-H groups on the alumina surface [52,53], and the weak electrostatic interactions of p-NP with the different sites of HDTMA^+^/Al_2_O_3_. Z. Wu et al. [47] found that molecular p-NP can be adsorbed on graphene oxide at lower pH by hydrophobic interaction and interactions of π-π with the aromatic fractions of p-NP and the aromatic matrix of graphene. Liu et al. [54], in their study of the adsorption of p-NP and phenol on an organometallic adsorbent, attributed the remarkable adsorption affinity for p-NP to the hydrogen bond between the OH or NO_2_ groups of p-NP and the amino groups NH_2_ of the adsorbent. Danish Khan et al. [49], in their molecular dynamics study of p-NP adsorption on kaolinite surface in aqueous medium, concluded that there were hydrogen bond-dominated interaction mechanisms for p-NP, than electron donor-acceptor and π-π interactions mechanisms [48].

**Figure 11:**
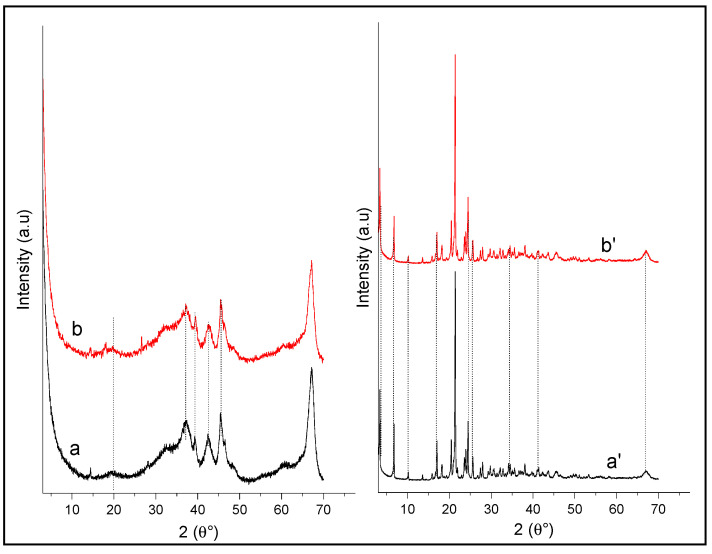
XRD patterns of (**a**) Al_2_O_3_ and (**b**) p-NP/Al_2_O_3_ and (**a’**) HDTMA^+^/Al_2_O_3_ and (**b’**) p-NP/HDTMA^+^/Al_2_O_3_.

On the other hand, the diffractogram of alumina alone remain unchanged after adsorption of p-NP (Figure 11a), which may be due to the interaction of p-NP with surface sites inside the adsorbent pores, since all the peaks observed at 2θ = 37.5° (220), 39.50° (311), 42.52° (222), 45.70° (400), and 67.30° (440) kept their positions even after p-NP adsorption. Identical observations can be made in the case of the adsorption of p-NP on the composite (Figure 11b), which indicates that the initial structures of the adsorbents do not change after adsorption of p-NP. However, the absence of the alumina peaks in the diffractogram of the composite is due to the adsorption of the polymer on its surface.

### 3.6. Antibacterial Activities

**Figure 12:**
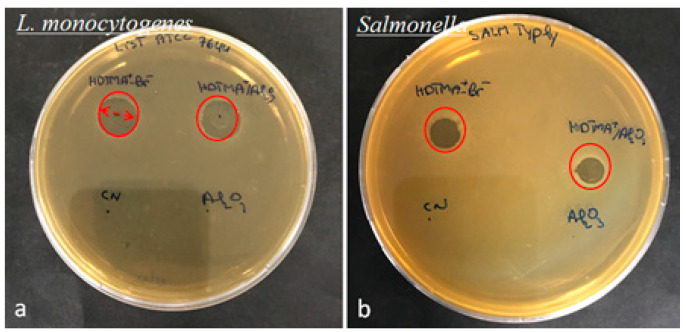
Inhibition zone of HDTMA^+^-Br^−^ and HDTMA^+^/Al_2_O_3_ against (**a**) *Listeria monocytogenes*, and (**b**) *Salmonella* spp.

The antibacterial activities of different concentrations of HDTMA^+^-Br^−^, Al_2_O_3_, and HDTMA^+^/Al_2_O_3_ composite suspensions, in the range of 0.05, 0.1, 0.5, 1, and 5 mg/L, were tested against different strains of *Salmonella* spp. and *Listeria monocytogenes*, by disk diffusion and broth microdilution methods. The results obtained from the disk diffusion assay showed that the diameters of the inhibition zone of materials ranged from 6 ± 0.1 to 24 ± 0.1 mm and from 8 ± 0.1 to 20 ± 0.1 mm against *Salmonella* spp. and *Listeria monocytogenes* strains, respectively (Table 5 and Figure 12). The Al_2_O_3_ revealed no inhibition zone against all tested *Salmonella* spp. and *Listeria monocytogenes* strains. Moreover, the diameters of the inhibition zone increased with increasing concentrations of materials. On the other hand, the broth microdilution assay showed that HDTMA^+^-Br^−^ and HDTMA^+^/Al_2_O_3_ displayed the best MIC values, ranging from 0.01 to 0.05 mg/mL, depending on *Salmonella* spp. and *Listeria monocytogenes* strains (Table 6). Thus, these materials exhibited a bactericidal activity against all *Salmonella* spp. and *Listeria monocytogenes* strains with the same MBC/MIC value of 1. Previous studies have shown that alumina (Al_2_O_3_) alone has a significant antibacterial effect against Gram-positive bacteria and has no antibacterial effect against Gram-negative bacteria [55,56]. Siamak Zavareh et al. [57] showed that chitosan biopolymer acts on the alteration of the Gram-negative bacteria wall, due to the presence on its surface of positive charges, which leads to a dysfunction of the bacterial metabolism. However, Cu-chitosan/nano-Al_2_O_3_ has an antibacterial activity 2.5 times greater than that of chitosan and 20 times greater than that of chitosan/nano-Al_2_O_3_ [58]. Moreover, Cu-chitosan/nano-Al_2_O_3_ has a more significant antibacterial activity against Gram-negative bacteria than Gram-positive bacteria [57]. Furthermore, HDTMA^+^-Br^−^ showed an antibacterial effect against Gram-positive bacteria [59,60]. Pritam Borker et al. reported that the positive charge of Zn^2+^ and Al^3+^ interacts with the cell wall of bacteria, binding to the surface of bacteria through a mechanism of electrostatic interaction. This produces reactive oxygen species on the cell wall surface, which leads to cell damage and cell death [61,62].

## 4. Conclusions

Our study shows that alumina before and after interaction with HDTMA^+^-Br^−^ has significant adsorption capacities and was particularly effective for the removal of p-nitrophenol. The equilibrium time of p-NP adsorption on Al_2_O_3_ and HDTMA^+^/Al_2_O_3_ at the temperatures studied was within 3 h. It was found that the adsorption rate of p-NP on both adsorbents increases with adsorption temperature and the presence of HDTMA^+^ polymer on the alumina surface improves the adsorption capacity of the composite with respect to the p-NP pollutant. The kinetic data were presented using the pseudo-second order kinetic model. The parameters suggested that the adsorption process of p-NP onto the adsorbents occurred via a physisorption process (activation energy < 40 kJ/mol) and was spontaneous (ΔG° < 0) and endothermic in nature (ΔH° < 0). The experimental p-NP adsorption isotherms can be described either by the Langmuir and Freundlich models. Hydrogen bonding can be the main mechanism affecting the adsorption capacity.

The results also showed that these samples possess good antibacterial activity against the different strains of *Salmonella* spp. and *Listeria monocytogenes* tested, with bactericidal effect for HDTMA^+^-Br^−^ and HDTMA^+^/Al_2_O_3_.

## Figures and Tables

**Table 2:** Kinetic parameters of linear modeling and the intra-particle diffusion model equations of p-NP adsorption at different temperatures onto Al_2_O_3_ and HDTMA^+^/Al_2_O_3_.

**Model**	**Sample**		**Al_2_O_3_**			**HDTMA^+^/Al_2_O_3_**		
	q_exp_ (mg/g)		1.480	1.820	2.000	2.940	3.150	3.210
	T (°C)		25	35	25	35	25	35
Lagergren	k_1_ (min^−1^)		0.016	0.017	0.018	0.011	0.007	0.010
	q_e_ (mg/g)		1.209	1.303	1.453	0.448	0.400	0.261
	$R_{1}^{2}$		0.885	0.790	0.760	0.453	0.165	0.106
	χ^2^		0.348	0.526	0.609	0.608	0.649	1.121
	SD		1.728	1.931	2.088	1.600	1.380	2.304
Kannan	${k^{\prime }}_{1}^{2}$ (min^−1^)		76.943	80.738	64.605	2.934	2.538	2.342
	q_t_ (mg/g)		2.121	2.815	2.878	2.907	3.063	3.183
	${R\prime }_{1}^{2}$		0.988	0.954	0.943	0.971	0.930	0.969
	χ^2^		1.536	2.381	2.077	0.062	0.080	0.047
	SD		0.793	0.636	0.500	0.065	0.055	0.048
Ho et Coll	k_2_ (g/mg min)		0.111	0.238	0.355	7.107	11.131	11.575
	q_e_ (mg/g)		1.730	2.055	2.201	2.911	3.039	3.199
	$R_{2}^{2}$		0.986	0.982	0.986	0.998	0.997	0.999
	χ^2^		0.005	0.003	0.002	2 10^−5^	2 10^−4^	2 10^−6^
	SD		0.060	0.050	0.040	0.004	0.010	0.001
Weber et Morris	Step 1	k_d1_ (mg/g)min^−1^	0.137	0.183	0.203	0.498	0.544	0.554
		C_i_	0.008	0.009	0.007	0.306	0.348	0.382
		$R_{1}^{2}$	0.966	0.964	0.967	0.860	0.846	0.837
		SD	0.013	0.018	0.019	0.113	0.130	0.137
	Step 2	k_d2_ (mg/g)min^−1^	0.021	0.016	0.013	0.013	0.011	0.012
		C_2_	1.116	1.521	1.733	2.657	2.837	2.967
		$R_{2}^{2}$	0.775	0.340	0.221	0.195	0.001	0.213
		SD	0.005	0.009	0.009	0.009	0.011	0.007

**Table 3:** Model parameters for the adsorption of p-NP on Al_2_O_3_ and HDTMA^+^/Al_2_O_3_ at different temperatures.

Sample		Al_2_O_3_			HDTMA^+^/Al_2_O_3_		
Model	T (°C)	25	35	45	25	35	45
Langmuir	q_m_ (mg/g)	6.186	4.032	3.929	12.690	9.966	8.919
	K_L_ (L/mg)	0.015	0.041	0.063	0.020	0.030	0.037
	R^2^	0.967	0.958	0.977	0.969	0.962	0.958
	χ^2^	0.019	0.023	0.018	0.022	0.026	0.023
Freundlich	K_F_ (mg/g)	0.101	0.213	0.33	0.292	0.369	0.416
	1/n	0.878	0.721	0.646	0.841	0.776	0.740
	R^2^	0.955	0.933	0.955	0.953	0.937	0.928
	χ^2^	0.022	0.015	0.024	0.014	0.023	0.012
Redlich-Peterson	K_R-P_	2 10^−5^	4 10^−6^	5 10^−4^	1 10^−4^	7 10^−7^	1 10^−10^
	A_R-P_	0.087	0.120	0.185	0.253	0.246	0.267
	α	3.222	3.898	2.47	0.262	0.426	0.721
	R^2^	0.991	0.972	0.989	0.969	0.989	0.992
	χ^2^	0.002	0.005	0.004	0.014	0.019	0.012

**Table 4:** Thermodynamic parameters for the adsorption of p-NP onto Al_2_O_3_ and HDTMA^+^/Al_2_O_3_.

Sample	Al_2_O_3_			HDTMA^+^/Al_2_O_3_		
T (°C)	25	35	45	25	35	45
E_a_ (kJ/mol)	7.848			10.252		
A (g.mg^−1^ min^−1^)	2.016			5.180		
R^2^	0.965			0.956		
SD	0.411			0.603		
ΔG° (kJ/mol)	−0.090	−0.285	−0.407	−0.727	−1.065	−1.261
ΔS° (J/K, mol)	15.924			26.839		
ΔH° (kJ/mol)	4.644			7.249		
R^2^	0.963			0.942		
SD	0.251			0.4887		

**Table 5:** Determination of the diameter of the inhibition zone of materials against (S) *Salmonella* spp. and (L) *Listeria monocytogenes* strains.

Sample	C_i_ (mg/mL)	Diameter of the Inhibition Zone (mm)															
		S_1_	S_2_	S_3_	S_4_	S_5_	S_6_	S_7_	S_8_	L_1_	L_2_	L_3_	L_4_	L_5_	L_6_	L_7_	L_8_
Al_2_O_3_	5	ND	ND	ND	ND	ND	ND	ND	ND	ND	ND	ND	ND	ND	ND	ND	ND
HDTMA^+^-Br^−^	5	22	24	19	22	23	18	17	21	18	16	16	16	14	20	16	17
	1	15	14	14	16	15	15	14	14	16	14	15	15	13	16	12	14
	0.5	7	6	6	9	9	12	14	7	14	14	15	14	10	14	12	14
	0.1	8	11	10	9	9	13	ND	8	13	13	12	12	10	13	9	13
	0.05	ND	ND	ND	ND	ND	ND	ND	ND	9	11	13	10	9	10	9	11
HDTMA^+^/Al_2_O_3_	5	18	22	15	20	22	12	13	20	15	13	15	14	12	15	15	14
	1	11	12	14	12	16	15	15	17	15	14	15	15	13	14	13	14
	0.5	8	9	8	9	8	15	10	6	13	11	14	12	10	12	11	11
	0.1	6	9	8	7	11	8	ND	8	12	11	12	12	9	11	9	11
	0.05	ND	ND	ND	ND	ND	ND	ND	ND	10	9	9	8	8	9	8	10

**Table 6:** Determination of MIC and MBC of HDTMA^+^-Br*^−^* and HDTMA^+^/Al_2_O_3_ against (S) *Salmonella* spp. and (L) Listeria *monocytogenes* strains.

Sample	C_i_ (mg/mL)	S_1_	S_2_	S_3_	S_4_	S_5_	S_6_	S_7_	S_8_	L_1_	L_2_	L_3_	L_4_	L_5_	L_6_	L_7_	L_8_
HDTMA^+^-Br^−^	CMI	0.1	0.5	0.1	0.1	0.1	0.05	0.1	0.1	0.01	0.01	0.05	0.01	0.05	0.01	0.05	0.01
	CMB	0.1	0.5	0.1	0.1	0.1	0.05	0.1	0.1	0.01	0.01	0.05	0.01	0.05	0.01	0.05	0.01
	R	1	1	1	1	1	1	1	1	1	1	1	1	1	1	1	1
HDTMA^+^/Al_2_O_3_	CMI	0.1	0.5	0.1	0.1	0.5	0.5	0.1	0.5	0.01	0.01	0.01	0.01	0.5	0.5	0.01	0.5
	CMB	0.1	0.5	0.1	0.1	0.5	0.5	0.1	0.5	0.01	0.01	0.01	0.01	0.5	0.5	0.01	0.5
	r	1	1	1	1	1	1	1	1	1	1	1	1	1	1	1	1

## Data Availability

Not applicable.
